# Significance of Cytomegalovirus gB Genotypes in Adult Patients Undergoing Hematopoietic Stem Cell Transplantation: Insights from a Single-Centre Investigation

**DOI:** 10.3390/ph17040428

**Published:** 2024-03-27

**Authors:** Tamara Vasiljevic, Marko Jankovic, Ana Tomic, Ida Bakrac, Stefan Radenovic, Danijela Miljanovic, Aleksandra Knezevic, Tanja Jovanovic, Irena Djunic, Milena Todorovic-Balint

**Affiliations:** 1 Faculty of Medicine, University of Belgrade, 8 Dr Subotica Street, 11000 Belgrade, Serbia; vasiljevict76@gmail.com (T.V.); ida.bakrac9@gmail.com (I.B.); radenovic_stefan@hotmail.com (S.R.); danijela.karalic@med.bg.ac.rs (D.M.); aleksandra.knezevic@med.bg.ac.rs (A.K.); irenadjunic04@gmail.com (I.D.); bb.lena@gmail.com (M.T.-B.); 2 Department of Virology, Institute of Microbiology and Immunology, 1 Dr Subotica Street, 11000 Belgrade, Serbia; 3 Institute for Biocides and Medical Ecology, 16 Trebevicka Street, 11000 Belgrade, Serbia; tanja.jovanovic@biocidi.org.rs; 4 Clinic of Haematology, University Clinical Centre of Serbia, University of Belgrade, 2 Dr Koste Todorovica Street, 11000 Belgrade, Serbia

**Keywords:** cytomegalovirus, gB genotype, hematopoietic stem cell transplant, adult, GvHD

## Abstract

Introduction: Cytomegalovirus (CMV) infection is a major clinical issue after allogeneic hematopoietic stem cell transplantation (HSCT). The CMV envelope glycoproteins are key in viral pathogenesis; the glycoprotein B (gB) encoded by the *UL55* gene might be an important determinant of viral virulence and disease severity marker in patients treated with allogeneic HSCT. Our aim was to investigate the molecular diversity of CMV gB and inquire into the associations between *UL55* gene variations and clinical manifestations in adult patients treated with allogeneic HSCT. Results: The most prevalent genotypes were gB1 and gB4 (11/27, 40.7%). Patients with genotype gB1 infection had earlier platelet engraftment (*p *< 0.033) and less frequent minimal/measurable residual disease post HSCT than those without this genotype. Patients with gB4 glycoprotein infection had a significantly lower CD4+/CD8+ ratio at D90 (*p *< 0.026). Interestingly, patients with gB5 glycoprotein infection had shorter overall survival from base condition diagnosis (*p *< 0.042), as well as shorter overall survival after HSCT (*p *< 0.036). Acute GvHD was noted more frequently in those with mixed-genotype infection (*p *= 0.047). Material and Methods: The study included fifty-nine adult patients treated with allogeneic HSCT. Peripheral venous blood was sampled typically per week, with detection of CMV performed by quantitative real-time PCR. Multiplex nested PCR was used to determine specific gB genotypes, which were then statistically compared vis-à-vis specific clinical variables. Conclusions: Our study points to variations in the viral *UL55* locus imparting both beneficial (earlier platelet engraftment, less frequent MRD post HSCT) and adverse effects (shorter overall survival, more frequent acute GvHD, less frequent 100% chimerism at day 90) to the transplanted host. Comprehensive molecular investigations are necessary to validate this apparent duality, as the potential benefits of CMV could perhaps be utilized for the benefit of the patient in the future.

## 1. Introduction

Cytomegalovirus (CMV), also known as human betaherpesvirus 5, is a DNA pathogen that falls under the *Orthoherpesviridae* family [1]. This versatile virus possesses a large genome of approximately 236 kb, housing over 200 protein-coding open reading frames. It exhibits significant genetic diversity with polymorphisms distributed across its genome [2]. CMV has the capability to replicate in various cell types, including hematopoietic, epithelial, and endothelial cells, as well as fibroblasts and smooth muscle cells [3].

Primary CMV infection typically occurs in early childhood and is often asymptomatic or may manifest occasionally as a mononucleosis-like syndrome [4]. However, in immunocompromised individuals, CMV can lead to severe diseases such as encephalitis, pneumonitis, hepatitis, uveitis, retinitis, and gastrointestinal issues. Notably, CMV is a frequent cause of congenital infections and a common contributor to central nervous system complications in newborns, such as mental retardation, cerebral palsy, and seizures [5]. In the context of immunocompromised patients, CMV infection poses a significant challenge, particularly after allogeneic hematopoietic stem cell transplantation (HSCT), resulting in considerable morbidity and mortality [6].

Similar to all herpesviruses, CMV establishes a lifelong latent infection after primary infection, which may sporadically reactivate. The virus’s genetic diversity allows for reinfection by different viral strains within the same host [3,4]. The genes encoding CMV envelope glycoproteins and play a crucial role in this diverse genetic assortment. These glycoproteins are involved in viral entry and cell fusion, serving as important targets for virus-neutralizing antibodies [3]. Surface glycoprotein B (gB), encoded by the *UL55* gene, is classified into four major genotypes (gB1, gB2, gB3, and gB4) and three non-prototypic ones (gB5, gB6, and gB7) [3,7,8,9,10,11,12,13]. Previous studies have emphasized gB’s essential role in virus ingress, cell-to-cell spread, and its potential significance as a virulence determinant [3,14].

Allogeneic HSCT is associated with severe treatment-related complications, including infections, high transfusion requirements, and graft-versus-host disease (GvHD) [15]. CMV stands out as a prominent pathogen affecting immunocompromised patients. Given that both the immune response and clinical outcomes of CMV infection may hinge on the viral strain [6], our objective was to explore the molecular diversity of CMV glycoprotein B and investigate associations between *UL55* gene variations and clinical manifestations in adult patients undergoing allogeneic HSCT.

## 2. Results

We investigated the molecular diversity of CMV glycoprotein B and its relation to clinical manifestations in adult patients treated with allogeneic HSCT. The study included fifty-nine patients (29 female, 30 male).

The incidence of CMV DNAemia was 44/59 (74.6%). Five gB genotypes (gB1, gB2, gB3, gB4, and gB5) were identified in the patient cohort. Out of 44 CMV positive samples, 40 were available for further genotypization (23 female, 17 male). Twenty-seven samples were successfully identified as gB1–gB5 genotypes. The remaining samples did not show the presence of these genotypes, suggesting the possibility that they might be positive for gB6 and/or gB7 genotypes, undetectable by the employed primers. Alternatively, it could indicate an unsuccessful genotyping process.

In total, in all CMV-positive patient samples combined, there were 46 genotype occurrences. The gB4 and gB1 genotypes predominated, being detected in samples of 11/27 (40.7%) patients each. Mixed genotype infection, when more than one gB variant was observed, was confirmed in 15/27 patients (55.5%).

Concerning the distribution of mixed gB variants, they appeared in the following combinations: five patients (gB3 + gB4), three patients (gB1 + gB2), two patients (gB1 + gB3), two patients (gB1 + gB2 + gB4), one patient (gB4 + gB5), one patient (gB1 + gB3 + gB5), and 1 patient (gB2 + gB4). The two-genotype combination predominated (12/15, 80%), while three genotypes appeared in 3/15 (20%) patients. The most prevalent variant in the mixed genotype complement was gB4 (9 instances out of 34; 26.5%), followed by gB1 and gB3 (8/34; 23.5%), and gB2 (6/34; 17.7%) and gB5 (3/34; 8.8%). Please note that the percentage of gB variants is compared to the complete number of genotypes observed (34 instances), rather than the total number of patients (15 patients with mixed genotypes), in order to visualize the frequency of genotypes vis-à-vis one another.

**Figure 1:**
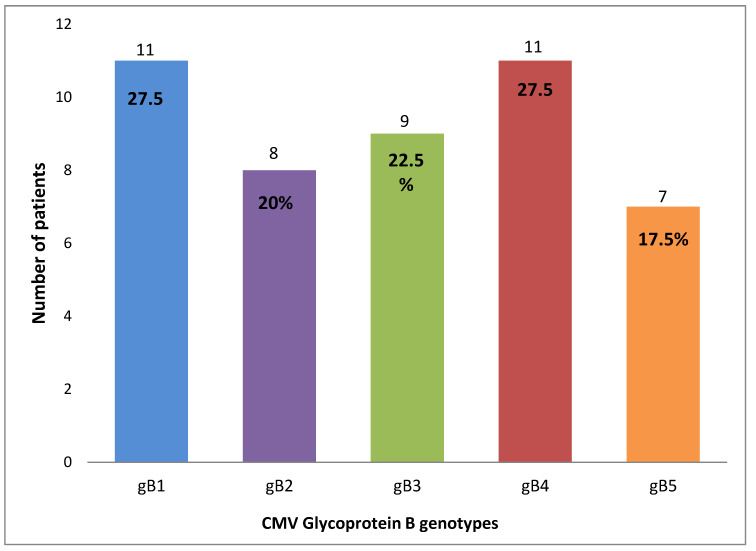
The histogram represents CMV glycoprotein B frequencies in allo-HSCTed patients.

**Figure 2:**
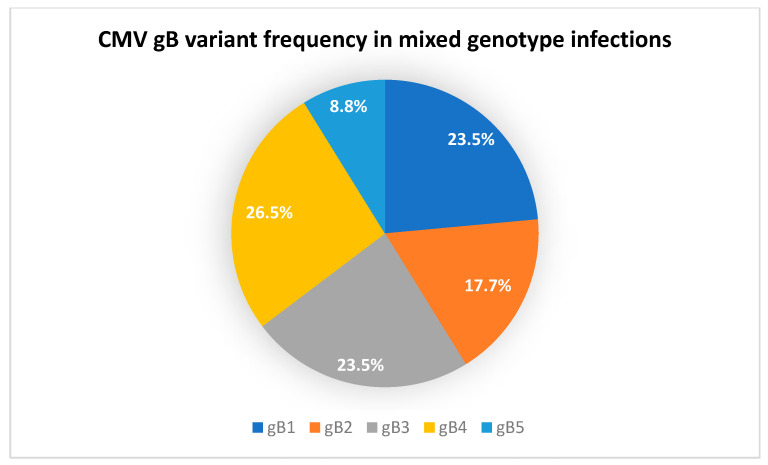
The chart represents the frequencies of CMV gB variants in patients with mixed infections.

All genotype frequencies can be observed in Figure 1 and Figure 2.

In patients with confirmed CMV infection, chronic GvHD occurred less often, albeit not significantly (*p *= 0.106). Conversely, poor graft function (PGF) was noted significantly more frequently in patients with CMV infection (*p *< 0.025) (Table 1).

Patients with genotype gB1 infection had platelet engraftment earlier than those without this genotype (*p *< 0.033). Minimal/measurable residual disease (MRD) post HSCT occurred less frequently in these patients (*p *< 0.011). Patients presenting with gB3 genotype infection achieved 100% chimerism less at D90 (*p *< 0.015). Patients with gB4 glycoprotein infection had a significantly lower CD4+/CD8+ ratio at D90 (*p *< 0.026).

Notably, patients with gB5 glycoprotein infection had shorter overall survival from base condition diagnosis (*p *< 0.042), as well as shorter overall survival after HSCT (*p *< 0.036). The same correlation was observed as regards mixed genotype infection, wherein patients with more than one gB genotype also had shorter overall survival from diagnosis (*p *< 0.036) and shorter overall survival after HSCT (*p* < 0.042); moreover, acute graft versus host disease was noted more frequently in this population (*p *< 0.047).

As presented in Table 2, an analysis of ULR was conducted, showing mixed genotype as a significant, independent variable for both OS from diagnosis, as well as OS from HSCT. After adjusting for age, type of transplant, relapses after alloHSCT, and previous acute GvDH in MLR, mixed genotype remained as a significant predictor for shorter OS from diagnosis and HSCT.

Statistically significant correlations as regards CMV infection, respective genotypes, and clinical parameters are presented in Table 1, Table 3, Table 4, Table 5, Table 6, Table 7, Table 8 and Table 9.

## 3. Discussion

To the best of our knowledge, this is the first study in Serbia that investigated the clinical significance of cytomegalovirus gB genotypes in adult patients treated with HSCT.

Human cytomegalovirus glycoprotein B is the best-characterized CMV glycoprotein to date. It is a prerequisite for HCMV entry into target cells and infection via cell-to-cell spread; moreover, it is a highly genetically variable protein [16] and a prime target for neutralizing antibodies.

To date, seven variants (four major: gB1, gB2, gB3, and gB4, and three non-prototypic: gB5, gB6, and gB7) were discovered. The first five genotypes have been detected in Asia, Europe, and North America. However, their geographic distribution differs: gB1 is the most prevalent genotype in Asia and Egypt [6,14,17,18,19,20,21,22,23,24,25,26,27,28,29,30,31,32,33,34,35,36,37,38]; gB1 and gB2 are frequently detected in North America [39,40,41,42,43,44,45]; in South America, the most frequent genotypes were gB1 and gB2 [9,10,46,47,48,49,50,51,52,53,54] whereas gB1, gB2, and gB3 are commonly observed across Europe (with the exception of Serbia where gB4 is the most prevalent genotype) [55,56,57,58,59,60,61,62,63,64,65,66]. In the present study, the most prevalent genotypes were gB1 and gB4, followed by gB3, gB2, and gB5 (Figure 1). This is in accordance with our previous results obtained from pediatric patients that underwent HSCT, wherein the gB1 and gB4 variants were the most predominant as well [67].

Cytomegalovirus (CMV) is a highly pervasive and ubiquitous herpesvirus. The pathogen’s prevalence rises to up about 100% in both Africa and Asia, and 80% in Europe and North America [68]. In the Republic of Serbia, the occurrence of CMV infection in patients with hematological B cell malignancies was found to be 90.4% [55]. Interestingly, in the same study, the age/gender matched non-tumor controls were significantly more pervaded by CMV (98.7%). The work by Zuhair et al. shows a significant swathe of CMV prevalences across Europe, ranging anywhere from 39% (Ireland) to 84% (Hungary) [69]. In the current study from a cohort of allo-HSCT-ed patients, the incidence of CMV infection was 74.6% (44/59). In Portugal, the overall prevalence of CMV infection in allogeneic hematopoietic stem cell transplantation (aHSCT) patients was 60.3% [70]. In a study by Kumar and colleagues, it was shown that the CMV reactivation rate was 43.8% amongst patients with aHSCT [71]. In Europe, the overall CMV seroprevalence in the adult population-based sample of Germany was 56.7% with a higher rate of seroprevalence in women (62.3%) than in men (51.0%) [72]. In solid-organ transplants, the incidence of CMV infection is 50% to 75% in patients undergoing heart–lung or lung transplantation and 50% in patients undergoing pancreas or kidney–pancreas transplantation. The incidence of CMV infection is 9% to 23% after heart transplantation and 22% to 29% after liver transplantation [73]. Yeh et al. demonstrated a high CMV seroprevalence rate in their study (92.2%) [74].

It is worthwhile noting that in our cohort, patients with CMV infection had a longer overall survival compared to those without (*p *= 0.037). Moreover, these patients developed chronic GvHD significantly less often (*p *= 0.049). Similar observations have been reported elsewhere; namely, in individuals who have undergone allogeneic HSCT, the reactivation of CMV has been linked to a marked decrease in the risk of leukemia relapse [75]. This finding is supported by the observation that rapid CMV replication may reduce the risk of relapse in non-Hodgkin lymphomas [76], acute myeloid leukemia [77,78,79], and pediatric acute leukemia following HSCT [80]. In a cohort of patients with myeloproliferative disorders, the reactivation of CMV after HSCT was associated with a slight reduction in the risk of early relapse [81]. These associations speak in favor of a potential CMV-vs.-malignancy effect, which was described in a number of studies.

When a gB genotype has been identified as a disease severity marker, it was often simply the most prevalent genotype in the study population [16]. A great number of studies have attempted to find a correlation between gB genotype and the occurrence of CMV-associated disease in immunocompromised patients [46,82,83]. Cytomegalovirus gB genotype may be an important determinant of viral virulence because gB has been implicated in several essential steps in CMV pathogenesis, such as virus entry, cell fusion, and cell-to-cell spread. The virulence of different CMV strains may be an important factor in the occurrence of CMV disease because of the genetic variation in genes that are involved in host cell penetration, tissue tropism, or replication, and polymorphism in the viral genome may play an important role [57,60,82]. Herein, we have observed several correlations between gB variants and clinical parameters of patients undergoing HSCT.

Compared to a single gB genotype infection, mixed gB genotype infections are frequently reported in organ/bone marrow transplant recipients and AIDS patients. Patients with mixed gB genotype infections have been reported to have faster disease progression or higher viral loads [11]. This study showed that acute GvHD more often presents in patients with mixed gB genotype infections, and these patients also show a shorter survival time from moment of diagnosis and/or transplantation.

CMV reactivation is the major infectious complication between 30 and 100 days after transplantation [84]. Reactivation of CMV appears in 60% of seropositive allo-HSCT recipients [84], which is somewhat less often than in our study where CMV incidence was found to be 74.6%. In this study, patients with CMV reactivation had acute GvHD more often, but not significantly so. Cytomegalovirus is associated with an increased incidence of opportunistic infections and GvHD in allo-HSCT recipients [76,85,86].

CMV infection is closely related to poor graft function (PGF) [87], which was also the conclusion in our study (*p *= 0.025). We also noted that CMV infection was not associated with chronic GvHD. The association between different CMV genotypes and different clinical characteristics of the transplant recipients has also been identified in other studies [6]. Madi et al. demonstrated that the gB1 genotype is significantly associated with development of fever, leukopenia, and severe CMV disease compared with other gB genotypes [82]. In a study by Rosen and colleagues on 53 CMV-infected liver transplant recipients, it was shown that gB1 genotype is significantly associated with a higher number of acute rejection episodes but not with the rejection severity [83]. In contrast, in a study on 58 liver transplant recipients with CMV infection, Sarcinella and colleagues showed that gB genotype does not correlate with peak CMV viral load, development of CMV disease, and acute rejection [39]. Within the scope of this investigation, we demonstrate that patients with the gB1 genotype manifest earlier platelet engraftment (*p *= 0.033) and less frequent minimal/measurable residual disease post HSCT (*p *= 0.011). This indicates that the gB1 variant may bestow some beneficial effects to the patient.

Viral gB3 and gB4 were reported to be associated with a lethal outcome because of myelosuppression in HSCT patients [40], and gB2 was associated with retinitis in AIDS patients [14]. In a report by Torok-Storb B et al., 21.3% of patients with gB types 3 and 4 died of infection associated with neutropenia compared with only 2.2% of patients with types 1 and 2. One could interpret this to mean either that gB types 3 and 4 are more likely to infect the marrow, or that types 3 and 4 are capable of escaping immune recognition in the marrow. Also, gB3 and gB4 were negatively associated with acute GvHD [40]. On the other hand, Dieamant et al. in their study found all gB3 genotypes were involved with acute GvHD, highlighting a possible association of that genotype with acute GvHD. The results of their study also showed that overall survival was 0% for gB3 and 57% for gB4 [46]. In our study, patients with the gB3 variant achieved 100% chimerism at day 90 post HSCT less often than those without (*p *= 0.015).

Finally, shorter OS from diagnosis and after HSCT was confirmed in patients with gB5 genotype infection. Notably, this association was not observed with other specific genotypes, hinting at a specific impinging effect of the gB5 genotype (or converse effect of multiple genotypes in concert) on patient survival. Although this genotype was not investigated in the study of Dieamant and coworkers [46], they reported on gB3 as the CMV variant with the least stratified overall survival (0%). To our knowledge, this study is the first to link the reduced lifespan post HSCT to the gB5 viral genotype.

## 4. Material and Methods

The study included 59 patients hospitalized in the Clinic for Hematology of the University Clinical Center of Serbia. The cohort comprised 29 (49.1%) female and 30 (50.9%) male patients treated with allogeneic HSCT for various hematological malignancies (Table 10). Informed consent was secured from all study participants. Ethical approval was obtained from the Ethical Committee of the Faculty of Medicine, University of Belgrade, Belgrade, Republic of Serbia.

The patients’ peripheral venous blood was sampled approximately once per week for the purposes of routine viral screening, with samples gathered from 2019 to 2021. Serially collected clinical samples were hence transported and analyzed at the virology laboratory of the Institute of Microbiology and Immunology, Faculty of Medicine, University of Belgrade, where they were tested via nucleic acid amplification techniques for the presence of CMV, Epstein–Barr virus (EBV), herpes simplex virus (HSV), human herpesvirus 6 (HHV-6), and BKV. Detection and quantitation of CMV was performed by quantitative real-time PCR on an ABI 7500 Real-Time PCR System (Applied Biosystems™, Thermo Fisher Scientific, Waltham, MA, USA).

After centrifugation, the buffy coat fraction underwent screening for CMV DNA, with subsequent genotyping of CMV-positive samples. Viral DNA was extracted from 200 μL of plasma utilizing the QIAamp^®^ Blood Mini Kit (Qiagen, Hilden, Germany) as per the manufacturer’s instructions. The preliminary detection and measurement of CMV DNA were executed through a TaqMan Real-Time PCR method. Specifically, primers were devised for amplifying a segment from the CMV immediate-early (*IE*) region gene, with the sequences 5′-CGC TCA CAT GCA AGA GTT AAT CTT C-3′ and 5′-AAC TCG GTA AGT CTG TTG ACA TGT ATG-3′. Additionally, the TaqMan probe was labelled with fluorescence at the 5′ and 3′ ends using 6-carboxyfluorescein (FAM; a reporter dye) and 6-carboxytetramethyl-rhodamine (TAMRA) dye, respectively, and had the sequence 5′-FAM CTC TAT CTG ACA TAC ACA AGT AAA TCC ACG TCC CA TAMRA-3′ [88]. The quantification standards employed in the real-time PCR procedure comprised plasmids containing viral amplicons for the CMV immediate-early antigen (IEA) region (Clonit, Italy). The range of the standards varied from 10^3^ to 10^6^ copies per µL. Determination of quantities was accomplished through the utilization of a standard curve calculation based on the Ct (threshold cycle) value standards. Values extrapolated from this curve were represented in copies/µL. To convert these values to copies/mL in the sample material and to adjust for sample dilution, multiplication by 150 was necessary.

For the detection of specific gB genotypes, we employed a multiplex nested PCR technique following a previously described methodology [11]. In essence, primers were designed to amplify specific sections of the *UL55* gene. Following the initial PCR round, the resulting amplicons underwent a subsequent multiplex step to determine the genotype. As a control, a PCR mixture containing RNAase-free water was used in place of the sample. Details of the primer sequences and conditions for the multiplex nested PCR reactions can be found in Table 11.

Each of the gB genotypes were readily identifiable on gel electrophoresis, as the exact genotype depended on fragment length. The DNA fragments were separated via electrophoresis in a 3% agarose gel, which was stained with SYBR™ Safe (Invitrogen™, Thermo Fisher Scientific, Waltham, MA, USA) and observed using an ultraviolet transilluminator. To facilitate the identification of amplicons, a size marker of 100 base pairs was included.

The clinical data were obtained from patient records gathered during hospitalization and outpatient visits to the Clinic for Hematology of the University Clinical Center of Serbia.

For data analysis, IBM^®^ SPSS^®^ Statistics v20 software was utilized. Univariate (ULR) and multivariate linear regression (MLR) analysis were performed in order to determine if mixed genotype was a significant predictor for shorter overall survival (OS) from time of diagnosis and HSCT. Variables deemed significant by previous tests were considered for inclusion in the model, along with expert opinion that helped define the number and order in which the explanatory variables were entered into the model. Wilcoxon–Mann–Whitney, Fisher’s exact test, and the Phi method were employed. A significance threshold of *p*-value <0.05 was established.

## 5. Limitations

We must concede to several limitations of our study. Firstly, our patient cohort consisted of a relatively limited number of individuals, and undoubtedly, a larger group would produce more accurate findings. The CMV reactivation incidence further reduced the number of CMV positives even more, which could have influenced the conclusions of the study. It is important to acknowledge that the genotype frequencies are also somewhat low, which has the potential to obfuscate genuine findings. Lastly, although the study length in itself is not a limiting factor, a longer follow up for each of the patients might have generated more precise results.

## 6. Conclusions

The association between CMV genotypes and specific clinical manifestations in patients treated with HSCT is still not fully elucidated, and a definitive link between a specific viral variant and clinical parameter remains to be established. Our study points to CMV genotypes imparting both beneficial (earlier platelet engraftment, less frequent MRD post HSCT) and adverse effects (shorter overall survival, more frequent acute GvHD, less frequent 100% chimerism at day 90) to the transplanted host. Variations in the viral *UL55* locus significantly associate with MRD post HSCT, earlier platelet engraftment, incomplete chimerism at day 90, and shorter survival from time of transplantation and time of diagnosis. Mixed gB infections emerge again as detrimental to the patient, being associated with shorter survival and acute GvHD. Furthermore, identification of gB genotypes that may be detrimental to the patient could perhaps signal an early therapy modification and close follow-up of said patients. Ultimately, the genotype(s) posing the highest risk to the patient may be evaluated for genotype-specific antibody therapy or the development of vaccines. Conversely, variants that appear to confer advantageous effects on the patient, particularly in terms of virus-vs.-cancer outcomes, may be investigated for potential targeted anti-tumor therapies. The association between gB genotypes and clinical outcomes implies potential usefulness of the gB protein as a prognostic marker—however, more detailed studies on larger cohorts are warranted and might yield more precise results.

## Figures and Tables

**Table 1:** Relevant associations between CMV reactivation and clinical parameters in allo-HSCT-ed patients.

Patients with CMV Infection			
*p* < 0.05	*p*-Value	*p* > 0.05	*p*-Value
Longer OS from Diagnosis	0.037	Longer OS after HSCT	0.088
Higher CD4+ at D360 (rel. value)	0.031	Longer time from Dg to HSCT (months)	0.093
Higher NK-Ly count at D180 (abs. value)	0.042	Higher Ly count at D360 (rel. value)	0.086
Less often have 1st complete remission	0.049	Higher NK-Ly at D180 (rel. value)	0.071
More often have PGF	0.025	More often have acute GvHD ^‡^	>0.05

OS—overall survival; PGF—poor graft function; HSCT—hematopoietic stem cell transplant. ^‡^ Tested independently for skin, GI tract and liver aGVHD.

**Table 2:** Regression analysis for mixed genotype as an independent predictor for given dependent outcomes.

Parameters	Univariate (Enter Method)		Multivariate (Forward Method) *	
	95% Confidence Interval	*p*-Value	95% Confidence Interval	*p*-Value
Shorter OS from diagnosis	−31.907; −1.426	0.034	−32.318;−0.635	0.042
Shorter OS after HSCT	−26.526; −2.274	0.022	−26.408; −1.354	0.032

* Adjusted for age, type of transplant, relapses after alloHSCT and previous acute GvHD. OS—overall survival; GvHD—graft-vs-host disease; HSCT—hematopoietic stem cell transplant.

**Table 3:** Associations between CMV gB1 genotype and specific clinical parameters.

Patients with gB1 Genotype Infection			
*p* < 0.05	*p*-Value	*p* > 0.05	*p*-Value
Earlier platelet engraftment	0.033	Lower CMV PCR max. copy/mL no. before D100	0.061
Lower Le at D360 (abs. value)	0.037	Lower OS from Diagnosis	0.073
Lower Ly at D360 (abs. value)	0.037	Lower B CD19+ at D180 (rel. value)	0.062
Lower CD8+ at D180 (rel. value)	0.04	Lower NK-Ly at D180 (rel. value)	0.089
Less often MRD post-HSCT	0.011		
Lower CD8+ at D360 (abs. value)	0.04		
Lower B CD19+ at D360 (abs. value)	0.037		
Lower NK-Ly at D30 (abs. value)	0.044		
Higher NK T-Ly at D90 (rel. value)	0.009		
Higher NK T-Ly at D90 (abs. value)	0.039		
Lower NK T-Ly at D180 (abs. value)	0.034		
Lower NK-Ly at D360 (abs. value)	0.037		

MRD—minimal/measurable residual disease; OS—overall survival; HSCT—hematopoietic stem cell transplantation.

**Table 4:** Associations between CMV gB2 genotype and specific clinical parameters.

Patients with gB2 Genotype Infection			
*p* < 0.05	*p*-Value	*p* > 0.05	*p*-Value
Lower CD4+ at D180 (abs. value)	0.048	Lower Ly at D180 (rel. value)	0.09
Lower NK-Ly at D30 (abs. value)	0.035	Lower Ly at D180 (abs. value)	0.066
Younger at time of HSCT	0.026	Lower CD4+ at D30 (abs. value)	0.092
		Lower CD8+ at D180 (abs. value)	0.066
		Lower NK T-Ly at D360 (rel. value)	0.079

HSCT—hematopoietic stem cell transplant.

**Table 5:** Associations between CMV gB3 genotype and specific clinical parameters.

Patients with gB3 Genotype Infection			
*p* < 0.05	*p*-Value	*p* > 0.05	*p*-Value
Lower B CD19+ at D30 (rel. value)	0.039	Lower Le at D30 (abs. value)	0.076
Less achieve 100% chimerism at D90	0.015	Lower Ly at D180 (abs. value)	0.068
		Lower CD4+/CD8+ at D90	0.069
		Lower CD4+ at D360 (rel. value)	0.088

**Table 6:** Associations between CMV gB4 genotype and specific clinical parameters.

Patients with gB4 Genotype Infection			
*p* < 0.05	*p*-Value	*p* > 0.05	*p*-Value
Lower CD4+/CD8+ at D90	0.026	Shorter OS from Diagnosis	0.09
		Lower CD4+/CD8+ at D30	0.051
		Lower CD4+ at D90 (rel. value)	0.055
		Lower CD4+ at D90 (abs. value)	0.072
		Lower B CD19+ at D360 (rel. value)	0.083
		Lower B CD19+ at D360 (abs. value)	0.053

OS—overall survival.

**Table 7:** Associations between CMV gB5 genotype and specific clinical parameters.

Patients with gB5 Genotype Infection			
*p* < 0.05	*p*-Value	*p* > 0.05	*p*-Value
Shorter OS from Diagnosis	0.042	Lower CD34+ (abs. value)	0.088
Shorter OS after HSCT	0.036	Lower Le at D30 (abs. value)	0.098
		Lower CD4+/CD8+ at D30	0.081

OS—overall survival; HSCT—hematopoietic stem cell transplantation.

**Table 8:** Correlations between diverse CMV genotypes (more than one) and specific clinical factors.

Patients with Mixed Genotype Infection			
*p* < 0.05	*p*-Value	*p* > 0.05	*p*-Value
Shorter OS from Diagnosis	0.036	Lower B CD19+ at D360 (abs. value)	0.053
Shorter OS after HSCT	0.042	Lower NK-Ly at D30 (rel. value)	0.059
More often have acute GvHD	0.047	Lower NK-Ly at D30 (abs. value)	0.065
Lower Ly at D90 (rel. value)	0.027	More often have an unfavourable outcome	> 0.05
Lower CD4+ at D90 (rel. value)	0.035		
Lower CD4+ at D90 (abs. value)	0.046		
Lower CD4+/CD8+ at D90	0.024		
Lower CD4+ at D180 (rel. value)	0.031		

OS—overall survival; HSCT—hematopoietic stem cell transplantation; GvHD—graft-vs-host disease.

**Table 9:** The relation between genotypes and variables related to post-transplant outcomes.

Genotypes	Relapse after Initial Therapy	Relapse after Allo-HSCT	Non-Relapse Mortality	Outcome (Alive/ Deceased)	Acute GvHD	Chronic GvHD	OS from Diagnosis	OS from HSCT
gB1	*p *= 0.505	*p *= 1.000	*p *= 0.796	*p *= 0.393	*p *= 0.608	*p *= 0.272	*p *= 0.215	*p *= 0.075
gB2	*p *= 1.000	*p *= 0.388	*p *= 0.886	*p *= 0.393	*p *= 0.126	*p *= 0.272	*p *= 0.324	*p *= 0.524
gB3	*p *= 0.258	*p *= 0.447	*p *= 0.387	*p *= 0.615	*p *= 1.000	*p *= 1.000	*p *= 0.569	*p *= 0.393
gB4	*p *= 0.580	*p *= 0.022 ^†^	*p *= 0.310	*p *= 0.085	*p *= 0.126	*p *= 1.000	*p *= 0.340	*p *= 0.408
gB5	*p *= 1.000	*p *= 0.067	*p *= 0.101	*p *= 0.051	*p *= 1.000	*p *= 1.000	*p *= 0.042 ^†^	*p *= 0.036 ^†^

OS—overall survival; GvHD—graft-vs.-host disease; HSCT—hematopoietic stem cell transplant. **^†^** *p*-values marked with the dagger symbol are significant.

**Table 10:** Basic patient demographics as regards CMV DNA detection.

Patient Characteristics		CMV DNA Positive	CMV DNA Negative	*p*-Value
Cohort size (*N*)	59	44	15	N/A
Avg. age at alloHSCT, range (year) Male Female	39.85 (19–64) 41.8 (20–58) 37.83 (19–64)	40.36	38.33	*p *= 0.554 *
Gender				*p *= 0.824 ^†^
Male	30	22 (73.3%)	8 (26.7%)	
Female	29	22 (75.9%)	7 (24.1%)	
Diagnoses				*p *= 0.739 ^†^
Acute lymphoblastic leukemia	16	11 (68.8%)	5 (31.2%)	
Acute myelogenous leukemia	28	22 (78.6%)	6 (21.4%)	
Hodgkin’s disease	7	4 (57.1%)	3 (42.9%)	
Myelodysplastic syndrome	5	4 (80%)	1 (20%)	
Non-Hodgkin’s lymphoma	2	2 (100%)	0	
Chronic lymphocytic leukemia	1	1 (100%)	0	
alloHSCT modality				*p *= 0.055 ^†^
MUD	23	20 (87%)	3 (13%)	
MMUD	18	13 (72.2%)	5 (27.8%)	
MRD	6	2 (33.3%)	4 (66.7%)	
HAPLO	8	5 (62.5%)	3 (37.5%)	
MMUD and MUD	4	4 (100%)	0	
CMV prophylaxis (letermovir)				*p *= 0.593 ^‡^
Yes	5	3 (60%)	2 (40%)	
No	54	41 (76%)	13 (24%)	
Acute GvHD				*p *= 0.260 ^†^
Yes	17	14 (82.4%)	3 (17.6%)	
No	37	25 (67.6%)	12 (32.4%)	
GvHD—skin	12	9 (75%)	3 (25%)	*p *= 0.875 ^†^
GvHD—liver	2	2 (100%)	0	*p* > 0.05 ^‡^
GvHD—gastrointestinal	7	6 (85.7%)	1 (14.3%)	*p *= 0.393 ^†^
Chronic GvHD				*p *= 0.106 ^†^
Yes	14	8 (57.1%)	6 (42.9%)	
No	43	34 (79.1%)	9 (20.9%)	

Abbreviations: N/A—not applicable; MUD—matched unrelated donor; MMUD—mismatched unrelated donor; MRD—matched related donor; HAPLO—haploidentical; GvHD—graft-vs-host disease. * Independent samples *t*-test value. ^†^ Pearson chi-square value. ^‡^ Fisher’s exact test value.

**Table 11:** Primer sequences and nested multiplex PCR conditions used in CMV gB genotyping.

Gene UL55	Primer	Primer Sequence (5′–3′)	PCR Conditions						Amplicon Size (bp)
			ID	D	A	E	FE	Cycles	
1st round	UL55 up	TTTGGAGAAAACGCCGAC	95/5 min	94/1 min	50/1 min	72/1 min	72/10 min	40	751
	UL55 low	CGCGCGGCAATCGGTTTGTTGTA							
2nd round	gB 1	ATGACCGCCACTTTCTTATC	94/2 min	94/30 s	53/1 min	72/1.5 min	72/10 min	35	420
	gB 2	AATTCGGTCTTCCAAAGTCGGAG							613
	gB 3	TAGCTCCGGTGTGAACTCC							190
	gB 4	CGAGTCCTCGGCTTCGGAACGAATGGT							465
	gB 5	GTTCTCCAGCGATAGGGTA							139
	gB low	GTTGATCCACACACCAGGC							

ID—initial denaturation, D—denaturation, A—annealing, E—elongation, FE—final elongation. All temperature values are expressed in degrees centigrade.

## Data Availability

All data are either publicly accessible or available from the corresponding author upon reasonable request.
